# Effect of Calving Difficulties and Calf Mortality on Functional Longevity in Polish Holstein-Friesian Cows

**DOI:** 10.3390/ani11102792

**Published:** 2021-09-24

**Authors:** Małgorzata Morek-Kopeć, Andrzej Zarnecki, Ewa Ptak, Agnieszka Otwinowska-Mindur

**Affiliations:** 1 Department of Genetics, Animal Breeding and Ethology, University of Agriculture in Krakow, al. Mickiewicza 24/28, 30-059 Krakow, Poland; ewa.ptak@urk.edu.pl (E.P.); agnieszka.otwinowska@urk.edu.pl (A.O.-M.); 2 Department of Cattle Breeding, National Research Institute of Animal Production, Krakowska 1, 32-083 Balice, Poland; rzzarnec@cyf-kr.edu.pl

**Keywords:** dairy cattle, difficult calving, stillbirth, longevity, survival analysis

## Abstract

**Simple Summary:**

Longevity, or the length of a cow’s productive life, is important in terms of profitability, animal welfare and environmental sustainability. In genetic evaluations, interest focuses on functional longevity, defined as a cow’s ability to avoid forced culling, an ability that increases the possibility of voluntary disposal based on economic criteria. Longevity is affected by several non-productive functional traits, among them those related to calving performance: calving ease (dystocia) and perinatal calf mortality (stillbirth). Parturition is a critical event in a cow’s life that has a number of different short- and long-term consequences. In the Polish Holstein-Friesian population, the incidence of dystocia and stillbirth is within the lower range of frequencies found in other dairy cattle populations. Our research showed that both traits affect functional longevity. Difficult calvings occur more frequently in heifers and increase the risk of involuntary culling more than in later parturitions. Additionally, a higher risk of culling is related to birth of a male calf. Moreover, the negative impact of calf mortality on longevity is also more pronounced in primiparous cows and in the case of delivery of male calves. Reducing the incidence of calving problems and perinatal mortality may improve the longevity of dairy cows.

**Abstract:**

Longevity is one of the functional traits that considerably affect dairy herd profitability. A Weibull proportional hazards model was used to evaluate the impact of difficult calvings and calf stillbirths on cow functional longevity, defined as length of productive life corrected for milk production. The data for analysis comprised calving ease and calf mortality scores of 2,163,426 calvings, 34.4% of which came from primiparous cows. The percentage of male calves was 53.4%. Calving ease was scored as “without assistance” (34.44%), “with assistance” (62.03%), “difficult—hard pull” (3.39%), and “very difficult, including caesarean section” (0.14%). Calf mortality scores were “live born” (94.21%) and “stillborn or died within 24 h” (5.79%). The Weibull proportional hazards model included classes of calving ease or calf mortality scores × parity (1, ≥2) × sex of calf as time-dependent fixed effect. The model also included time-dependent fixed effects of year × season, parity × stage of lactation, annual change in herd size, fat yield and protein yield, time-independent fixed effect of age at first calving, and time dependent random herd × year × season. In first-parity cows, very difficult birth of a bull or heifer increased the relative risk of culling, respectively, 2.18 or 1.26 times as compared with calving without assistance. In later parities, the relative risk of culling related to very difficult calving was 2.0 times (for male calves) and 1.33 times (for female calves) higher than the relative risk of culling associated with calving without assistance. Calf mortality showed a negative impact on longevity in both heifers and cows. First-parity stillbirth increased the relative risk of culling depending on sex of calf by 18% in females and by 15% in males; in later parities the increase of the relative risk of culling was lower (by 7% for females, 9% for males). Difficult calvings and their consequences, especially in primiparous cows, may negatively influence dairy herd profitability by reducing the length of cows’ productive life.

## 1. Introduction

Longevity is considered to be one of the most important traits influencing dairy herd profitability, and it is also strongly linked with animal welfare and environmental sustainability [1,2,3,4]. Until the late 1970s, selection in Holstein-Friesian populations was focused on production traits. This led to spectacular progress in milk yield, but at the same time caused a decline in non-yield functional traits [5]. Several studies have shown antagonistic genetic correlations between milk production and functional traits [6,7]. A decline in cow health and fertility, along with calving problems, resulted in higher rates of forced culling, consequently shortening the average productive life of cows. Reduced longevity has had an adverse effect on herd profitability [5,8,9,10].

To reverse the negative trend in longevity, selection goals have been changed and management practices have been improved. In the majority of dairy-producing countries, the selection indexes now include longevity and other functional traits [4,11,12]. Longevity is defined in several ways; its terminology and definitions have been reviewed by Schuster et al. [3] and Dallago et al. [2]. In studies on dairy cow lifespan, two definitions have been used most frequently: true longevity, that is, the ability to delay culling; and functional longevity, a cow’s ability to delay involuntary culling that is caused by, for example, health or fertility problems [13]. Functional longevity is approximated by adjusting survival for individual deviation from the within-herd production level, permitting the assumption that it is independent of voluntary culling based on breeder decisions. Improvement of functional longevity helps reduce forced culling and thus offers an opportunity to increase the voluntary culling rate. From the economic point of view, it allows breeders to make replacement decisions that are optimal, but do not necessarily result in longer lifespan [4,14].

Survival analysis is a methodology commonly used in genetic evaluations of longevity. In this approach, survival is treated as a continuous variable, measured as the number of days from first calving to culling, death, or censoring. However, it is not the lifespan itself, but the likelihood of culling that is modeled, and when adjusted for the effect of milk yield it represents functional longevity. The model of choice in that approach is the Weibull proportional hazard model, which accounts for a non-normal distribution of longevity data and allows inclusion of records of still-living cows (i.e., censored records) as well as time-dependent environmental effects [13,15,16,17,18,19].

Survival analysis can examine the effect of other functional traits on longevity, taking into account their variability over time. Studies based on this methodology have demonstrated the significant influence of several reproduction, type, health, and workability traits on length of productive life [20,21,22,23,24,25,26]. Among the traits negatively affecting longevity are calving ease and calf stillbirth [27,28,29,30].

In Holstein-Friesian populations, the frequency of difficult calvings ranges from 3% to 13%, and it is higher in the first parity when compared with later parities [31,32,33,34,35,36]. In primiparous cows, loss in yield traits was found to increase with the severity of dystocia; in later parities, significant depression of yield was observed only in the case of extreme calving difficulty [37]. Dystocia caused a significant reduction in yield traits and had a negative effect on a wide spectrum of dams’ physiological functions, especially fertility traits such as calving interval, number of services, days to first service, and nonreturn rate to 56 days [37,38].

Stillbirth frequency follows a pattern similar to that of frequency of difficult calving, with a higher rate of calf mortality observed at first calvings than in later parturitions. In most studies, the stillbirth rate for heifers ranged from 4% to 17%. In later calvings, the stillbirth rate varied between 2% and 10% [39,40,41,42,43,44,45,46].

A high percentage of difficult calvings is reflected in a higher risk of forced culling due to depressing effects on milk, fat and protein yields, days open, number of services, and cow losses. The consequences of dystocia and stillbirth directly and indirectly reduce herd profitability [8,10,37].

The aims of this study were: (1) to examine the incidence of calving ease and perinatal calf stillbirth, and (2) to assess the impact of calving ease and perinatal calf mortality on the functional longevity of Polish Holstein-Friesian cows.

## 2. Materials and Methods

### 2.1. Longevity Data

Longevity records of Polish Holstein-Friesian (HF) cows based on test-day dates, 305-day lactational yield, dates and codes of disposal were extracted from the Polish National Milk Recording System, SYMLEK database. Cows of all parities were included. Data edits included removing records with incorrect calving or culling dates, imposing a restriction of 18–48 months on cow age at first calving, and excluding herds with less than 15 cows and sires with less than 15 daughters. Length of productive life (LPL) of a cow was calculated as number of days from first calving to culling or censoring. A lifetime record was considered to be uncensored (completed) if the cow had a culling code (except “sold for dairy purposes”). Otherwise, records of cows still alive were regarded as censored (incomplete). Functional longevity was defined as the ability of a cow to avoid involuntary culling, and was approximated by LPL corrected for within herd × year × season production level [15,16]. The final longevity data set included records for 1,734,002 cows from 19,098 herds, which calved for the first time between 1995 and 2012. The cows were daughters of 10,077 sires. The average length of productive life of 863,076 cows (49.8%) with uncensored survival records was 1097.6 days, and maximum LPL was 6089 days. Mean censoring time for the remaining 870,926 cows was lower, amounting to 959.8 days. Maximum censoring time was 6253 days.

### 2.2. Calving Data

Based on calving ease and calf mortality scores reported by breeders, then collected by the Polish Federation of Cattle Breeders and Dairy Farmers (PFCBDF) and transferred to SYMLEK database, the following four categories of calving ease (CE) were defined: unassisted calving (1 = “without assistance”), calving with assistance (2 = “with assistance”), difficult calving with the use of considerable force (3 = “difficult”), and very difficult calving, including embryotomy and caesarean-section (4 = “very difficult”). Two categories of calf mortality (CM) were defined: calf born alive and survived the first 24 h (1 = “alive”), and calf stillborn or died within 24 h (2 = “stillborn”). Calving data were restricted to single calvings with known sex of calf. Cows were categorized according to parity as primiparous (parity 1) or multiparous (parities 2 to 6). The resulting data set consisted of 2,163,426 calvings recorded in 2006–2012.

Cows with longevity data and without information on calving performance or calf mortality, grouped in separate subclasses, were included in the analysis to reduce the bias [20].

### 2.3. Statistical Models

The influence of calving difficulty and perinatal mortality on the functional longevity of cows was analyzed using survival analysis [47]. Subclasses of interaction between calving ease (CE) or calf mortality (CM) categories and parity of dam (primiparous, multiparous) and sex of calf (male, female) were included as covariates in the following Weibull proportional hazard (PH) models:

Model M1:

h(t) = h_0_(t)exp[hys(τ) + age + ys(τ) + ls(t) + hsize(τ) + fat(t) + prot(t) + CEps(t)] (1)

Model M2:

h(t) = h_0_(t)exp[hys(τ) + age + ys(τ) + ls(t) + hsize(τ) + fat(t) + prot(t) + CMps(t)] (2)

where t is time from first calving to culling or censoring, τ is calendar time, h(t) is the hazard function (instantaneous probability of culling) for a cow at time t, h_0_(t) = λρ(λt)^ρ−1^ = ρt^ρ−1^exp(ρlogλ) is the baseline hazard function describing the natural ageing process, assumed to follow a Weibull distribution with scale parameter λ and shape parameter ρ [15].

The exponential term describes the factors influencing the risk of culling; some of these factors are changing with time.

The fixed effects included in the model were as follows: age—time-independent effect of monthly classes of age at first calving (<20, 21, 22, …, >40 months); ys—year × season comprising years 1995 to 2012 and 2 seasons (April–September, October–March), a time-dependent effect changing with each new season; ls—time-dependent combined effect of lactation number × stage of lactation, comprising the first five and pooled later lactations and four stages of lactation (1–29, 30–179, 180–304, >304 days of lactation), changing with each lactation date and new stage of lactation; hsize—yearly herd size variation with classes, reflecting the relative change of herd size from the current year to the next year (from <−50% to ≥50%; in 20% intervals), a time-dependent effect changing on 1 April each year; fat and prot—within-herd classes of 305-day fat and protein yield levels relative to herd mean, defined separately for the first and later lactations (from <−50% to ≥50%; in 10% intervals, and “no data” class), a time-dependent effect changing with new lactation date.

The fat and prot effects, representing the main reason for voluntary culling, were included in the model to approximate functional longevity [15,16].

CEps was the combined effect of CE × parity × sex of calf (a total of 16 classes, and class 17 = “no CE data”), CMps was the combined effect of CM × parity × sex of calf (8 classes in total, and class 9 = “no CM data”); both were time-dependent effects changing at calving date.

The random effect included was hys—time-dependent herd × year × season effect comprising years from 1995 to 2012 and 2 seasons: April–September and October–March; assumed to be independently distributed, following a log–gamma distribution; changing values on 1 April and 1 October each year.

The generalized coefficient of determination R^2^ of Maddala [48] was used to determine the proportion of total variation explained by the model:

R^2^Maddala = 1 − (L_0_/L_M_)^2/n^ (3)

where n is the sample size, L_0_ is the value of the likelihood function for a model with no predictors, and L_M_ is the likelihood for the model being estimated.

The significance and the overall influence of fixed effects on longevity were checked by a series of likelihood ratio tests comparing the full model with a reduced model excluding one effect under testing at a time. The changes in the log-likelihood function (−2logL) were analyzed to compare the impact of each covariate on risk of culling.

The random hys effect was algebraically integrated out, and the γ parameter of the hys distribution was estimated jointly with other effects.

Solutions for the fixed class effects, including CEps, CMps, were expressed as relative risks of culling (RRC). RRC represents the ratio between the estimated risk of culling caused by a specific level of a particular factor and the risk (set to one) for a chosen reference class with other factors assumed to be constant [49].

## 3. Results and Discussion

### 3.1. Dystocia and Stillbirth Incidence

Frequency of calving ease and calf mortality categories by parity of dam and sex of calf are presented in Table 1; 96.47% of the calvings fell in the “no assistance” and “with assistance” categories, and 3.53% were “difficult” or “very difficult”. The percentages of male and female calves were 53.4% and 46.6%, respectively; 34.4% of calvings were from primiparous cows. The frequency of calvings with assistance was higher in first (70.5%) than in later parities (57.6%). Difficult and very difficult calvings were more than twice more frequent in heifers (5.5% and 0.2%) than in cows (2.3% and 0.1%). The incidence of calving difficulties varied depending on sex of calf. Birth without assistance was more frequent for female calves (37.7%) than for males (31.6%). Cows giving birth to a bull calf required assistance more frequently (64.3%) than in the case of heifer calves (59.5%). The frequencies of difficult and very difficult births of male calves (4% and 0.2%, respectively) were about twice higher than the corresponding figures for female calves (2.7% and 0.08%). Sawa et al. [50] reported a similar overall incidence of calving difficulties and differences in dystocia rates associated with calf sex in the Polish Holstein-Friesian population. Pogorzelska and Nogalski [36] obtained results close to ours for overall dystocia rates, as well as a higher incidence of dystocia in heifers. Despite the differences between calving difficulty scoring systems used internationally (see Mee [31]; Zaborskiet et al. [51]), our results are also consistent with those observed in other populations with similarly defined traits. Overall incidence of about 3.5% for dystocia, and almost 6% frequency of dystocia in heifers, found in our study, were close to the lower values of the respective ranges of 3–13% and 3.1–23% reported for different Holstein-Friesian populations by Mee [31]. The higher dystocia rates in heifers as well as in the case of birth of male calves are in agreement with results presented in reviews by Mee [31] and Zaborski et al. [51].

As shown in Table 1, the incidence of calf perinatal mortality in our data was 5.8% overall, but differed depending on sex of calf and parity of dam. Almost twice higher was the frequency of stillbirth in heifers (8.2%) as compared with cows (4.5%). Similarly, the stillbirth rate of male calves (7.7%) was more than twice higher than for stillborn female calves (3.7%). For single births of Polish Holsteins, Sawa et al. [50] found the overall rate of perinatal mortality to be 7.5%, with mortality of male calves almost three times higher than for female calves. This slightly higher overall incidence of stillbirth could be at least partly associated with the higher proportion of first calvings in their data. Closer to our results are the mortality rates reported for the Polish population by Pogorzelska and Nogalski [36] (5.6% of all births) and Piwczyński et al. [52] (8.1% for first, 5.49% for second, 3.53% for third calving). Piwczyński et al. [52] also found a difference in the stillbirth rate between male (7.97%) and female (4.39%) calves. The calf mortality rate found in that study was rather low as compared with the wide range of results reported for other populations. According to Mee [53], the overall incidence of perinatal calf mortality in HF cattle varied from 4.3% (Ireland) to 9.6% (Canada), with higher values in heifers, ranging from 7.2% (Israel) to 16.6% (The Netherlands). Higher incidence of stillbirth for bull calves as well as first-calvers has also been reported in other studies [30,32,54,55,56].

**Figure 1:**
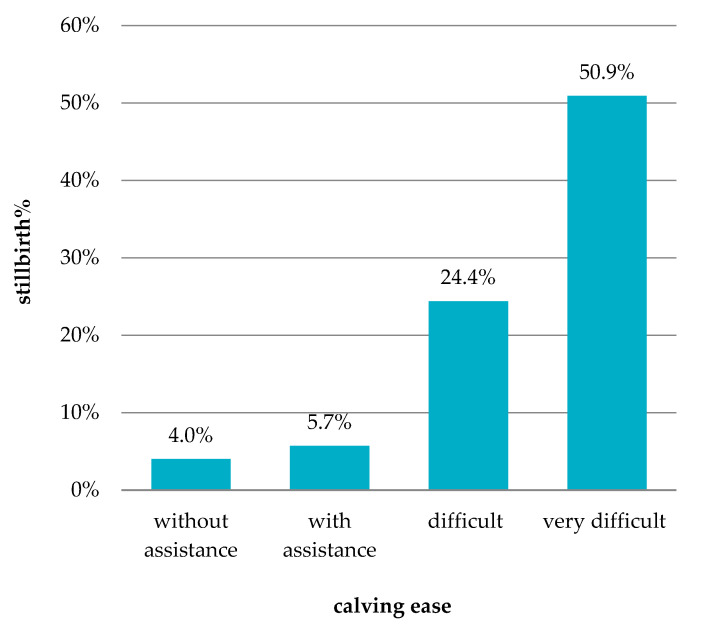
Percentage of stillbirth in calving ease categories.

In the literature, perinatal mortality in cattle is presented as the result of the interaction of multiple factors, with difficult calving reported as the primary cause, accounting for up to 50% of cases [57]. In our data, only 15% of all stillborn calves were born with difficulty (CE categories 3 and 4), 61% with assistance, and 24% without assistance (Table 2). Figure 1 shows how the stillbirth rate rises with increasing calving difficulty. While a relatively low percentage of stillbirths was recorded in the first two CE categories (4.0% in calving without assistance, 5.7% in calving with assistance), in the difficult and very difficult categories the stillbirth rate was much higher (24.4% and 50.9%, respectively). Pogorzelska and Nogalski [36] presented similar results for the Polish HF population. Yao et al. [54] observed stillbirth increasing with the frequency of calving difficulty in US Holsteins (from 4% for no difficulties to 44.3% for extreme difficulties) and Brown Swiss (from 4% to 39.4%).

In discussing the association between calving difficulties and calf mortality, some authors have pointed out that more than half of stillbirths concern calves born without any difficulties [58,59,60]. Reasons for stillbirth not related to dystocia, listed by Mee [31,61], and Barrier et al. [62], included placental dysfunction, low birth weight (especially in heifer calvings), heavier weight of male calves resulting in prolonged calving, body shape and length rather than birth weight, and longer and thinner body, which may be associated with inadequate prenatal development. Often, however, no obvious cause of death could be specified [57].

### 3.2. Survival Analysis

Two Weibull PH models were applied to the longevity data to analyze the effect of dystocia (model M1) and calf mortality (model M2) on length of productive life of dams. The estimates of the Weibull distribution parameters defining the baseline hazard function were almost the same for both models under study. In particular, for Model M1 the shape parameter ρ was 2.15 and the intercept (ρlogλ) was −10.9, which means increasing culling risk for animals as they age. Likewise, the same estimates were obtained from both models for the hys distribution parameter (γ = 1.8) and R^2^ of Maddala (0.67).

All fixed effects showed a highly significant impact on length of productive life (*p* < 0.0001). The combined effect of lactation number and lactation stage (Table 3) had the largest overall effect on hazard of culling (measured by −2logL change). Smaller impacts were found for protein production, annual herd size change, and year × season effects, and the influence of age at first calving and fat production was much lower. The above results are consistent with those published previously for the Polish HF cattle population by Morek-Kopec and Zarnecki [22]. They are also in the range of results reported for other cattle populations [49,63,64,65,66,67,68]). As shown in Table 3, the overall impact of calving ease and calf mortality on culling hazard was rather low as compared with other fixed effects. The changes in −2logL estimated for CEps (model M1) and CMps (model M2) effects were similar.

**Figure 2:**
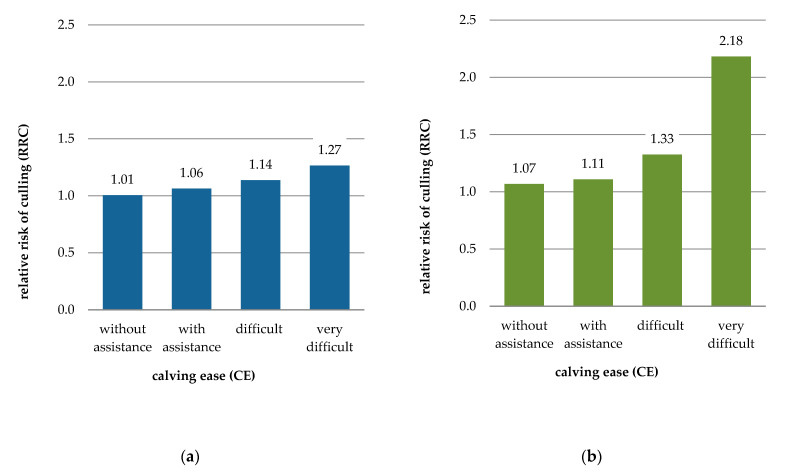

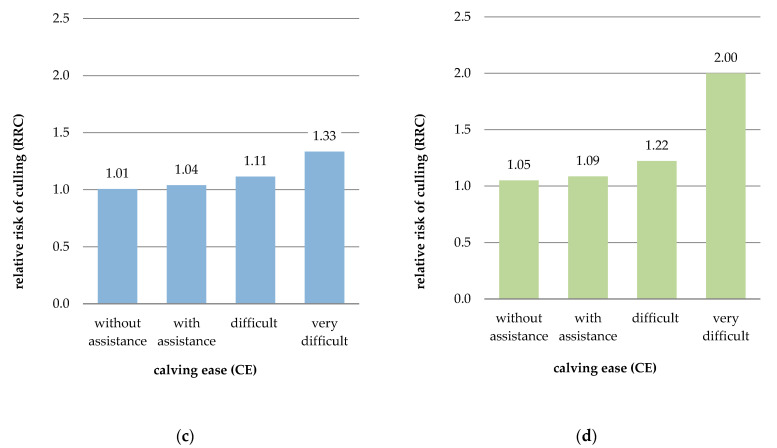
Relative risk of culling (RRC) for calving ease category (CE) by sex of calf and parity of dam; (**a**) female calf and primiparous cow, (**b**) male calf and primiparous cow, (**c**) female calf and multiparous cow, (**d**) male calf and multiparous cow.

The details of the influence of calving difficulty and calf mortality on functional longevity are presented in Table 4 and Table 5, showing the relative risk of culling associated with CE and CM categories by parity of dam and calf sex. Generally, regardless of parity of dam or calf sex, higher CE scores, indicating increased calving difficulty, resulted in higher RRC estimates (Table 4 and Figure 2). In primiparous dams, calving with assistance only slightly increased the risk of culling (6% for female calf, 4% for male) versus the respective calving without assistance. The corresponding increase of culling risk resulting from difficult calving was larger: 13% for a female calf and 24% for a male, and even larger in the case of very difficult calving: 26% for a female calf and 104% for a male. Similarly, the more difficult the calving, the higher the risk of culling in multiparous dams. The RRC associated with very difficult calving of male calves was almost twice that found for male birth without assistance (90% higher). The corresponding difference for female calvings was lower (30%). Relative to unassisted calving, difficult birth of male and female calves increased the culling risk of later-parity dams by 11% and 16%, respectively, and calvings requiring assistance increased it by 3% for both calf sexes.

The negative effect of difficult calving on dam longevity was more pronounced in primiparous cows. Especially in the case of male calves, any difficulties (including some assistance) occurring in the first parity were associated with culling risk higher than that resulting from similar problems in subsequent parities. In particular, the RRC for first calving of male calves classified as with assistance, difficult, and very difficult were, respectively, 2%, 8%, and 9% higher than RRC for similarly classified later calvings.

The first-parity calvings of cows delivering a female calf with assistance or with difficulty were also associated with higher culling risk than corresponding later-parity calvings. In both primiparous and multiparous cows the risk of culling associated with calving of male calves was generally higher than that for calving of female calves. In first-parity dams, very difficult delivery of a male calf resulted in a 1.7 times higher risk of culling as compared to a very difficult female birth, while in later-parity dams it was 1.5 times higher. In the category of difficult calving, the culling risk associated with male birth was higher than that associated with female birth by about 16% for the first parity and 10% for later parities, and in the category of calving with assistance by about 5%, regardless of parity.

In the “without assistance” category, the RRC estimates for male birth were also slightly higher (by 6% for first and 4% for later parity) than for female birth. In this case, the difference did not result from calving difficulties, but rather was associated with other factors related to calf gender.

Our present results agree with findings from other studies showing a negative impact of calving difficulties on functional longevity in dairy cattle. Using a Weibull PH model, Sewalem et al. [27] found that Canadian Holstein first-parity cows requiring hard pull or surgery were associated with about a 30% and 90% higher risk of being culled, respectively, as compared with cows calving unassisted. For Jerseys and Ayrshires, they reported an up to 50% increase of culling risk as a result of calving difficulties. Using a similar methodology, de Maturana et al. [10] investigated the impact of calving ease on functional longevity in Spanish Holsteins, based on data from primiparous and multiparous cows. They found an 18% overall increase of culling risk for calvings classified as dystocia as compared with unassisted or needing only slight assistance. The same authors reported that calving difficulty had a much greater impact on culling in the first parity than in later parities, expressed in up to twice higher RRC, depending on calving ease class. Based on data from large US Holstein dairy herds, De Vries et al. [28] showed that cows experiencing very difficult calving had about a twice greater risk of being culled than cows that had easy calving, and that first-parity cows having difficult calving were under a higher risk of culling than older cows. The negative influence of dystocia on cow longevity was also reported in studies based on other definitions of a trait representing longevity and using other statistical methods (e.g., Weller and Ezra [29]).

**Figure 3:**
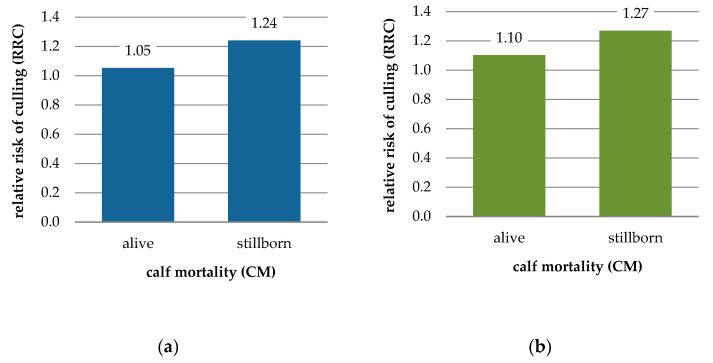

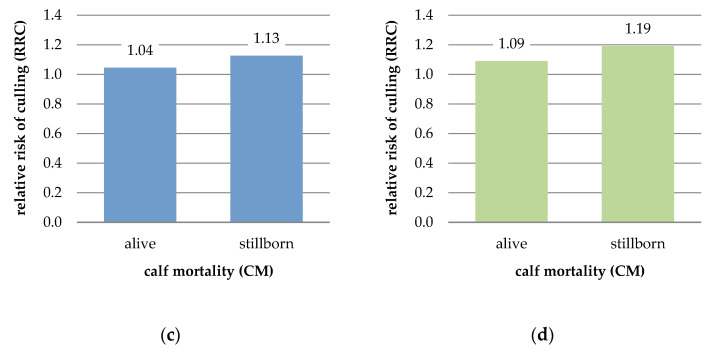
Relative risk of culling (RRC) for calf mortality category (CM) by sex of calf and parity of dam; (**a**) female calf and primiparous cow, (**b**) male calf and primiparous cow, (**c**) female calf and multiparous cow, (**d**) male calf and multiparous cow.

The estimates of relative risk of culling presented in Table 5 and Figure 3 show the negative impact of calf perinatal mortality on dam survival. In all subclasses of parity × sex of calf, stillbirth was associated with higher RRC as compared with normal calving: in primiparous dams by 18% for female calves and 15% for males, and in multiparous dams by 8% and 9%, respectively. The RRC estimates for stillbirth in the first parity were higher than the RRCs for stillbirth occurring in later parities: by 7% for a male birth and 10% for a female birth. The RRCs related to male stillbirth were generally slightly higher than these related to female stillbirth: by 2% in the first parity and 6% in later parities. The overall risk of culling resulting from stillbirth was highest for first-parity calving of male calves.

Our results showing the negative effect of calf mortality on dam longevity agree with results from other authors. In Canadian first-parity Holstein cows, Sewalem et al. [68] found a 15% greater culling risk for those delivering stillborn calves. Using a Weibull proportional hazards model to study the relationship between reproduction traits and functional longevity in Canadian Holstein, Ayrshire, and Jersey cows, Sewalem et al. [27] found a negative impact of stillbirth on dam survival; they observed a roughly 33% greater risk of being culled (average across three breeds) for cows giving birth to stillborn calves as compared with cows with normal parturition. Even higher risk estimates in US Holsteins were reported by Bicalho et al. [30], who also used survival analyses to assess the effect of stillbirth on dam survival. They found an increased culling risk for cows that had stillbirths, by almost 40% across all parities. Cows experiencing stillbirth are at higher risk of a number of postpartum disorders that may reduce their chance of survival [30].

In this study, differences in RRC estimates depending on sex of calf were also observed for the “alive” category (CM = 1, Table 5). Both primiparous and multiparous cows giving birth to a live male calf had a greater risk of being culled; it was about 5% higher than for those giving birth to a live female calf. Such a result, along with the already mentioned finding of an increasing effect of male calving on dam’s risk of culling even in the case of parturition without any difficulties (CE = 1; Table 4), supports the more general conclusion that birth of a male calf negatively affects cow longevity, regardless of calving problems. Analyzing different reproductive risk factors influencing culling and productive life in US Holsteins, De Vries et al. [28] also reported that cows giving birth to males were at a greater risk of culling than those having female calves, by 7%, 6%, and 5% in the 1st, 2nd and 3rd parities, respectively. In the literature, various reasons are given for male birth being more risky from the perspective of dam longevity. While some of them result in higher dystocia and stillbirth rates (e.g., heavier birth weight or the different conformation of male calves), others are independent of calving problems.

## 4. Conclusions

Dystocia and perinatal calf mortality are important components of cow reproductive performance. The overall frequency of difficult calvings and stillbirth in our studies was similar to that observed in other Holstein populations. Twice as many cases of dystocia were recorded in heifer parturitions than in later calvings. Similarly, the rate of calf stillbirth was twice higher in primiparous cows than in multiparous cows. The frequency of difficult calving and calf mortality was higher for delivery of male calves.

A Weibull proportional hazards model was used to evaluate the risk of forced culling associated with difficult calvings and stillbirth. All fixed effects included in the model showed a significant impact on length of productive life. The largest overall influence on risk of culling had combined effect of stage and number of lactation, and, in decreasing order, the effects of protein yield, change of herd size, year × season subclass, and age at first calving. Calving difficulties and calf mortality were also significantly associated with culling risk, which was higher in the case of heifer parturitions and delivery of male calves. Both traits negatively affected the length of production life in the Polish Holstein-Friesian cow population. Our results suggest that reducing the frequency of difficult calvings would positively affect functional longevity.

## Figures and Tables

**Table 1:** Frequency of calving ease and calf mortality categories, by sex of calf and parity of dam.

Trait	Class	Sex of Calf		Parity of Dam		Overall
		Male	Female	Heifer	Cow	
Calving ease (CE)	Without assistance	364,503	380,578	177,410	567,671	745,081
		31.56%	37.74%	23.82%	40.02%	34.44%
	With assistance	742,421	599,604	524,889	817,136	1,342,025
		64.28%	59.46%	70.47%	57.60%	62.03%
	Difficult	45,824	27,430	40,908	32,346	73,254
		3.97%	2.72%	5.49%	2.28%	3.39%
	Very difficult	2235	831	1636	1430	3066
		0.19%	0.08%	0.22%	0.10%	0.14%
Calf mortality (CM)	Alive	1,066,626	971,476	683,829	1,354,273	2,038,102
		92.35%	96.33%	91.81%	95.47%	94.21%
	Stillborn (or died within 24 h)	88,357	36,967	61,014	64,310	125,324
		7.65%	3.67%	8.19%	4.53%	5.79%
Overall		1,154,983	1,008,443	744,843	1,418,583	2,163,426
		100%	100%	100%	100%	100%

The frequencies differ significantly (*p* < 0.01) within sex, parity, calving ease and calf mortality.

**Table 2:** Distribution of calving ease categories in calf mortality subclasses.

Calving Ease (CE)	Calf Mortality (CM)	
	Alive	Stillborn
Without assistance	715,293	29,788
	35.10%	23.77%
With assistance	1,265,958	76,067
	62.11%	60.70%
Difficult	55,347	17,907
	2.72%	14.29%
Very difficult	1504	1562
	0.07%	1.25%
Overall	2,038,102	125,324
	100%	100%

**Table 3:** Results of a series of likelihood ratio tests (comparing full model with reduced models excluding one effect at a time), checking the significance and the overall influence of fixed effects on risk of culling.

Effect Excluded from Full Model	Model M1			Model M2		
	Δdf	−2logL Change	R^2^ of Maddala	Δdf	−2logL Change	R^2^ of Maddala
Lactation number × stage of lactation (ls)	23	401,290.0	0.5782	23	401,550.0	0.5781
Age at first calving (age)	21	3040.50	0.6647	21	2123.10	0.6649
Year × season (ys)	35	17,772.00	0.6619	35	17,811.00	0.6619
Relative fat yield (fat)	13	875.4	0.6651	13	991	0.6651
Relative protein yield (prot)	13	37,987.00	0.6579	13	38,172.00	0.6579
Herd size variation (hsize)	8	19,739.00	0.6615	8	19,879.00	0.6615
CE × parity × sex of calf (CEps)	16	1264.40	0.6651			
CM × parity × sex of calf (CMps)				8	1365.00	0.6651
Full model			0.6653			0.6653

Model M1: h(t) = h_0_(t)exp[hys(τ) + age + ys(τ) + ls(t) + hsize(τ) + fat(t) + prot(t) + CEps(t)], Model M2: h(t) = h_0_(t)exp[hys(τ) + age + ys(τ) + ls(t) + hsize(τ) + fat(t) + prot(t) + CMps(t)], df = degrees of freedom, −2logL change = likelihood ratio test statistics, asymptotically following a χ^2^ distribution with degrees of freedom equal to the difference in df (Δdf) resulting from excluding the effect tested. The changes in the log-likelihood function show the impact of each covariate on risk of culling. R^2^Maddala = 1 − (L_0_/L_M_)^2/n^ (where n is the sample size, L_0_ is value of the likelihood function for a model with no predictors, and L_M_ is the likelihood for the model being estimated) shows the proportion of total variation explained by each tested model. All effects highly significant (*p* < 0.0001).

**Table 4:** Relative risk of culling (RRC) and number of uncensored records (N) for CE category, by sex of calf for primiparous and multiparous cow (RRC = 1 for class “no CE data”).

Calving Ease (CE)	Primiparous Cow				Multiparous Cow			
	Female Calf		Male Calf		Female Calf		Male Calf	
	RRC	N	RRC	N	RRC	N	RRC	N
Without assistance	1.0053	11,389	1.0677	10,873	1.006	64,842	1.0505	67,963
With assistance	1.0636	31,404	1.1094	37,757	1.0383	81,307	1.0865	111,363
Difficult	1.1377	2535	1.3252	4119	1.1141	3128	1.223	6272
Very difficult	1.2662	110	2.1823	365	1.3327	169	2.0003	483

**Table 5:** RRC and number of uncensored records (N) for CM category, by sex of calf for primiparous and multiparous cow (RRC = 1 for class “no CM data”).

Calf Mortality (CM)	Primiparous Cow				Multiparous Cow			
	Female Calf		Male Calf		Female Calf		Male Calf	
	RRC	N	RRC	N	RRC	N	RRC	N
Alive	1.0525	42,458	1.1019	45,491	1.0447	144,230	1.0906	171,760
Stillborn	1.2399	2980	1.2692	7623	1.126	5216	1.191	14,321

## Data Availability

Restrictions apply to the availability of these data. Data was obtained from the Polish Federation of Cattle Breeders and Dairy Farmers.
